# Ideal vs. real: a systematic review on handling covariates in randomized controlled trials

**DOI:** 10.1186/s12874-019-0787-8

**Published:** 2019-07-03

**Authors:** Jody D. Ciolino, Hannah L. Palac, Amy Yang, Mireya Vaca, Hayley M. Belli

**Affiliations:** 1 Department of Preventive Medicine, Biostatistics Collaboration Center, Feinberg School of Medicine, Northwestern University, 680 N Lake Shore Drive, Suite 1400, Chicago, IL 60611-4402 USA; 2 AbbVie Inc, North Chicago, IL USA; 3 AY analytics, Chicago, IL USA; 4 AbbVie Inc, North Chicago, IL USA; 5 Department of Population Health, New York University Langone Health, New York, NY USA

**Keywords:** Randomization, Allocation, Stratification, Minimization, CONSORT

## Abstract

**Background:**

In theory, efficient design of randomized controlled trials (RCTs) involves randomization algorithms that control baseline variable imbalance efficiently, and corresponding analysis involves pre-specified adjustment for baseline covariates. This review sought to explore techniques for handling potentially influential baseline variables in both the design and analysis phase of RCTs.

**Methods:**

We searched PubMed for articles indexed “randomized controlled trial”, published in the *NEJM*, *JAMA*, *BMJ*, or *Lancet* for two time periods: 2009 and 2014 (before and after updated CONSORT guidelines). Upon screening (343), 298 articles underwent full review and data abstraction.

**Results:**

Typical articles reported on superiority (86%), multicenter (92%), two-armed (79%) trials; 81% of trials involved covariates in the allocation and 84% presented adjusted analysis results. The majority reported a stratified block method (69%) of allocation, and of the trials reporting adjusted analyses, 91% were pre-specified. Trials published in 2014 were more likely to report adjusted analyses (87% vs. 79%, *p* = 0.0100) and more likely to pre-specify adjustment in analyses (95% vs. 85%, *p* = 0.0045). Studies initiated in later years (2010 or later) were less likely to use an adaptive method of randomization (*p* = 0.0066; 7% of those beginning in 2010 or later vs. 31% of those starting before 2000) but more likely to report a pre-specified adjusted analysis (*p* = 0.0029; 97% for those initiated in 2010 or later vs. 69% of those started before 2000).

**Conclusion:**

While optimal reporting procedures and pre-specification of adjusted analyses for RCTs tend to be progressively more prevalent over time, we see the opposite effect on reported use of covariate-adaptive randomization methods.

**Electronic supplementary material:**

The online version of this article (10.1186/s12874-019-0787-8) contains supplementary material, which is available to authorized users.

## Background

It is generally agreed upon in the research community that a properly designed and implemented randomized controlled trial (RCT) serves as the optimal form of evidence-based research for establishing efficacy of a given therapy. The randomness element allows researchers the confidence that on average, study arms are similar and the only differing factor between these like groups is the intervention to be examined for efficacy. Statistically, this will allow for unbiased assessment of interventional effects with accuracy and precision. However, an individual trial must by definition exhibit some form of imbalance with respect to both measured and unmeasured confounders due to the random nature of the design. Although the expected level of imbalance is zero in these studies, no one trial will actually have zero imbalance on all (or any) important prognostic variables.

While an expected level of covariate imbalance not identically equal to zero may seem trivial, existing literature [1–5] illustrates the impacts of baseline variable imbalance on statistical parameters in analyses of intervention effects. Briefly, less than ‘statistically significant’ imbalance at the 5% level has the potential to impact power, type I error rate, and bias in marginal intervention effect estimates. The magnitude of these effects depends on the degree of association with outcome and both the directionality and magnitude of imbalance [1, 2, 4, 5]. Intuitively, if an interventional arm exposed to new therapy has a poorer disposition (e.g., increased disease severity at baseline) than a simultaneously measured placebo arm, it will be more difficult to detect a successful intervention effect if one exists. This translates into bias in an unadjusted treatment effect estimate and a corresponding loss of statistical power. Conversely, if that interventional arm has favorable prognosis in general at the beginning of the trial, it will be easier to claim the interventional arm has favorable outcome even if the new therapy is not efficacious; this corresponds to an increase in type I error rate.

Randomization literature and statistical theory literature have provided methodologies to mitigate these effects of baseline prognostic variables in both the design and analysis phases of RCTs. A common method for handling covariate imbalance is stratified block randomization. The idea of stratification and use of blocked randomization within strata dates to the mid-twentieth century [6, 7], and involves implementing separate pre-specified randomization sequences within subgroups of participants. While easy to both implement and understand, the randomization literature points to some faults in the methodology of stratified block randomization. Namely, the inability to handle large numbers of covariates/strata, the requirement to categorize continuous baseline variables, the requirement for pre-generated lists that may introduce additional sources of error if allocations become out of sequence, and the increased risk of selection bias when allocation becomes predictable.

To address the concerns of stratified block randomization, beginning in the 1970s, researchers developed a wide range of methods that fall under the general category of covariate-adaptive designs, or more simply “minimization”. Loosely defined, this is an adaptive allocation method that will strive to *marginally* (i.e., no longer within each stratum combination) balance several covariates at once [8]. The balance may be accomplished using some function (variance, range, etc.) to define “imbalance” for each variable of interest. An advantage of these covariate-adaptive designs lies in their flexibility of and range of choices for imbalance functions that can incorporate relative weights of covariates, more variables than stratified block methods, and continuous variables [9–12].

Despite ability to control baseline variable imbalance in an efficient and adaptive manner, employing such adaptive methods in a clinical trial often presents a logistical concern as they require complex algorithm implementation and programming with continual feedback, more extensive testing, and thus increased effort from the perspective of a trial programmer or statistician. It is generally agreed that investigators should attempt to control covariate imbalance (whether it be via stratification or covariate-adaptive methods), but adaptive methods carry more flexibility and better performance properties, resulting in “big rewards in scientific accuracy and credibility”. Despite the evidence suggesting the benefit of implementing covariate-adaptive designs, their use in modern day clinical trials remains limited. For example, in a review by Lin et al. 11–12% of trials examined reported the use of covariate-adaptive methods [13]. We speculate the complexity of covariate-adaptive designs may not be worth the added benefits to researchers. While software is available to implement such methods, these programs can be costly and their inputs not well understood, making interpretation of the randomization and subsequent results challenging. Further, the limited use of covariate-adaptive randomization techniques in practice may jeopardize the validity of findings across RCTs. As shown in the literature [1–5], seemingly small discrepancies across arms due to baseline covariate imbalance are propagated through to the final study analysis. In the era of reproducible research, imprecision due to covariate imbalance could lead to conflicting study results across repeated studies.

Related to study interpretation and reproducible research, in 2010, the Consolidated Standards of Reporting Trials (CONSORT) explanation was established to improve the clarity with which study methods are reported [14]. The CONSORT explanation recognizes the utility of restricted randomization that will control baseline variable imbalance, and it explains the benefits of stratification and minimization. It specifically highlights the limited number of variables that may be practical under stratification and the need for some random component applied to a minimization algorithm to prevent possible selection bias.

In adjusting for baseline variables in analyses, the CONSORT explanation further makes recommendations regarding appropriate vs. inappropriate adjustment in RCTs: “Although the need for adjustment is much less in RCTs than in epidemiological studies, an adjusted analysis may be sensible, especially if one or more variables is thought to be prognostic” [14]. Several authors have argued the benefits on increasing precision and reducing bias in various settings for known prognostics variables [5, 15–20]; however, CONSORT and International Conference on Harmonization (ICH) statements recommend this adjustment be pre-specified. Specifically, “the decision to adjust should not be determined by whether baseline differences are statistically significant” [14]. Taken together, there remains confusion and debate with regard to handling potentially influential baseline variables in RCTs [1, 3, 14, 21].

In summary, potentially influential baseline variables require special consideration in both the design and analysis phase of clinical trials. According to literature and guidelines, it would be ideal to control for these variables both at baseline, through stratified or adaptive allocation methods, and at analyses, with adjustment as appropriate. With these guidelines in mind, coupled with the most recent CONSORT explanation, we carried out a systematic review of published RCTs in four top tier journals with the ultimate goal of summarizing current practice in handling baseline variables in RCTs (i.e., the “real” world RCTs as opposed to the theoretical RCTs). Specifically, this review sought to (1) explore the frequency of use of allocation scheme types in published RCTs, and (2) explore the handling of prognostic covariates in analyses of clinical trial data. These results reveal not only the status of covariate adaptive techniques in modern RCTs, a measure important to adaptive research methodologists, but also the validity of such studies and their interpretability in the era of reproducible research.

## Methods

Since the most recent CONSORT guidelines went into effect in 2010, we chose to review articles published prior to 2010, and 4 years after (in 2014). Specifically, we searched PubMed for articles indexed with the publication type “randomized controlled trial”, published in the *NEJM, JAMA, BMJ,* or *Lancet* for the time periods of January 1, 2009 through June 30, 2009 and January 1, 2014 through June 30, 2014. Previously, Austin et al. [21] conducted a similar review in the *New England Journal of Medicine (NEJM)*, *Journal of the American Medical Association (JAMA)*, *Lancet*, and *British Medical Journal (BMJ)*. However, this review reflects just a single time period, and did not measure the changes after the CONSORT guidelines had gone into effect. The present work therefore not only builds on ideas from this previously published review, but also measures *changes* in the frequency of use of allocation scheme types in published RCTs, and explores *changes* in the handling of prognostic covariates in analyses of clinical trial data *over time*.

### Search criteria and screening

We employed the following search criteria: (randomized controlled trial[Publication Type] AND (“N Engl J Med”[Journal] OR “JAMA”[Journal] OR “BMJ”[Journal] OR “Lancet”[Journal]) AND ((“2009/01/01”[PDAT]: “2009/06/30”[PDAT]) OR (“2014/01/01”[PDAT]: “2014/06/30”[PDAT]))). We chose these specific journals to expand upon the previous review by Austin and colleagues [21], and further these journals historically carry high impact factors while reporting results across a diverse group of fields. We acknowledge that the review of articles within these four journals at these time periods restricts our sample and thus generalizability. However, we sought to review high-quality RCTs and this sample by nature includes articles screened through a highly-selective and rigorous peer review process.

Data for each article were housed in the Research Electronic Data Capture (REDCap) platform at Northwestern University [22]. We randomly assigned each article for screening to two study team members. Each member reviewed the abstract of the article to which she was assigned for screening to determine if the article should be included in full review. Exclusion criteria included: not an RCT, review paper, editorial/commentary/research letter, report on more than one clinical trial, and secondary analyses of an already published trial.

Upon consensus regarding inclusion for review and completion of the screening process, full review of each article proceeded with the goal of abstracting a list of pre-specified data elements (refer to Additional file 1). Full review included a complete review of the manuscript and Additional file 1 (e.g., statistical analysis plan, study protocol, previous published design papers) referenced in the manuscript. The review process followed a “first pass/second pass” pattern: one author (JDC) reviewed all articles passing screening and entered all available, relevant data into the data collection instrument housed in REDCap. She left the record as “unverified” in REDCap, and a second reviewer (MV, HLP, AY) performed a “second pass”, reviewing each article a second time and ensuring accuracy of data extraction and entry and indicating final agreement of data as “complete” in REDCap. In cases of discrepancies, the study team utilized the Data Resolution Workflow query system in REDCap to reach consensus.

### Variable extraction and analyses

We computed descriptive statistics summaries (frequencies and percentages) for the following outcomes:

1. Covariate involvement in randomization (yes vs. no/unable to determine)
2. Use of covariate-adaptive allocation methods (within subset of trials in #1)
3. Use of adjustment in analyses
4. Whether adjusted analyses were pre-specified (within subset of trials in #3)

Other variables extracted from each manuscript included: sample size, number of arms, number of study centers, whether the RCT was cluster-randomized, clinical trial type (superiority, non-inferiority, etc.), nature of primary outcome (continuous, binary, etc.), publication year/study initiation year, study length, and presence of a baseline test for significant difference in covariates (refer to Additional file 1).

We examined simple descriptive statistics (frequency [percentages] or median [interquartile range; IQR] as appropriate) for the variables listed above in aggregate and by publication year (2009 vs. 2014). Basic statistical tests (chi-squared and Wilcoxon Rank-Sum, as appropriate) examined association between reporting year (2009 vs. 2014) and relevant variables (including the outcomes listed above). In a secondary, exploratory series of analyses, we used series of individual simple logistic regression analyses to explore potential associations between study characteristics and each of the four aforementioned outcomes. That is, each logistic regression model included just one independent variable (i.e., we did not adjust for potential confounders).

There were no adjustments made for multiple hypothesis tests as these analyses were deemed exploratory in nature, and all tests assumed a 5% level of significance. Analyses utilized SAS (version 9.4, Copyright 2012 by SAS Institute Inc.; Cary, NC) and R (version 3.2.2, Copyright 2015 by The R Foundation for Statistical Computing Platform).

## Results

**Fig. 1:**
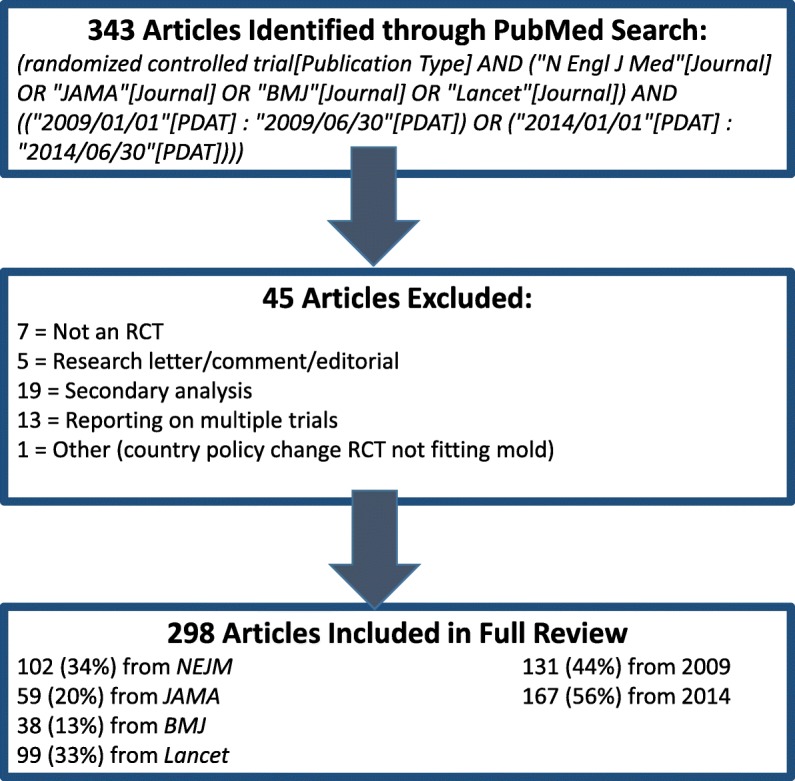
Article Search Results. Our search returned a total of 343 articles, 45 of which were excluded based on criteria illustrated here. Thus, the full review included 298 articles. Note that the one article excluded under the “Other” category was a non-typical RCT; the intervention was a country-wide policy change. Although it did not fall into the list of exclusion criteria, the reviewers made the post hoc decision to exclude this paper since the data could not be abstracted in the format we required for analyses

Our search returned 343 articles meeting inclusion criteria. Of these, 45 were excluded for the reasons illustrated in Fig. 1, resulting in a final review sample size of 298 RCTs. A slight majority (56%) of reviewed articles came from the publication year 2014.

### Trial characteristics

As shown in Table 1, most reviewed trials fell under the “superiority” heading (86%), and the majority (79%) were two-armed studies; the number of arms in all reviewed studies ranged from two to 24. An overwhelming majority (92%) were multicenter studies (with the number of centers as high as 1315) with a median 556 participants randomized. A small percentage (7%) employed a cluster-randomized design. Median reported study length was 3 years and participants involved in these studies were followed for a median of 12 months. It is important to reiterate that all statistical test results (*p*-values) were not adjusted for multiple hypothesis tests in analyses to follow as they were purely exploratory.

**Table 1:** Study Characteristics ^a^

Study Characteristic		Overall	2009	2014	*p*-value ^b^
Type of Study	Superiority	255 (86)	110 (84)	145 (87)	0.3047
	Non-Inferiority	23 (8)	9 (7)	14 (8)	
	Other	20 (7)	12 (9)	8 (5)	
Cluster-Randomized	No	277 (93)	117 (89)	160 (96)	0.0297
	Yes	21 (7)	14 (11)	7 (4)	
Number of Arms	2	236 (79)	107 (82)	129 (77)	0.5970
	3	24 (8)	8 (6)	16 (10)	
	4	26 (9)	12 (9)	14 (8)	
	5 or more	12 (4)	4 (3)	8 (5)	
Multicenter Study	Single Center	24 (8)	15 (11)	9 (5)	0.0563
	Multicenter	274 (92)	116 (89)	158 (95)	
Number of Centers		22 (5–71)	20 (4–68)	24 (6–76)	0.6291
Number of Subjects		556 (242–1408)	700 (279–1888)	457 (222–1224)	0.0770
Length of Study, Years (if reported)		3 (2–5)	3 (2–5)	3 (2–5)	0.6667
Months of Subject Follow-up (if reported)		12 (4–20)	12 (4–22)	12 (4–19)	0.8274
Study Initiation Year	Before 2000	17 (6)	13 (10)	4 (2)	<.0001
	2004–2005	75 (26)	66 (52)	9 (5)	
	2005–2009	123 (42)	48 (38)	75 (45)	
	2010 and later	78 (27)	0 (0)	78 (47)	
Primary Outcome	Continuous	89 (30)	42 (32)	47 (28)	0.2663
	Binary	113 (38)	42 (32)	71 (43)	
	Time-to-Event	84 (28)	40 (31)	44 (26)	
	Other	12 (4)	7 (5)	5 (3)	
Randomization Indicated in Title	No	94 (32)	40 (31)	54 (32)	0.7398
	Yes	204 (68)	91 (69)	113 (68)	
Allocation Method	Purely random allocation	4 (1)	3 (2)	1 (1)	0.1660
	Blocked (Permuted or Random block)	24 (8)	9 (7)	15 (9)	
	Stratified or Stratified block	205 (69)	82 (63)	123 (74)	
	Minimization/Covariate-adaptive method	32 (11)	18 (14)	14 (8)	
	Other	4 (1)	2 (2)	2 (1)	
	Unable to determine	29 (10)	17 (13)	12 (7)	
Covariate(s) Included in Allocation	No	27 (9)	12 (9)	15 (9)	0.3278
	Yes	241 (81)	102 (78)	139 (83)	
	Unable to determine	30 (10)	17 (13)	13 (8)	
Number of Covariates	1	95 (39)	42 (41)	53 (38)	0.5106
	2	86 (36)	32 (31)	54 (39)	
	3	40 (17)	17 (17)	23 (17)	
	4	11 (5)	5 (5)	6 (4)	
	5 or more	9 (4)	6 (6)	3 (2)	
Baseline Test for Significant Differences	No	167 (57)	76 (59)	91 (55)	0.5564
	Yes	126 (43)	53 (41)	73 (45)	
Unadjusted or Adjusted Analyses	Unadjusted	49 (16)	27 (21)	22 (13)	0.0100
	Adjusted	87 (29)	27 (21)	60 (36)	
	Both	162 (54)	77 (59)	85 (51)	
Reason for Adjustment (if applicable)	Pre-specified	226 (91)	88 (85)	138 (95)	0.0778^c^
	Data driven - Lack of balance	5 (2)	3 (3)	2 (1)	
	Data driven - Potential confounding	6 (2)	4 (4)	2 (1)	
	Other	4 (2)	3 (3)	1 (1)	
	Unable to determine	8 (3)	6 (6)	2 (1)	

^a^Percentages were calculated based on non-missing values

^b^*P*-value corresponds to chi-squared test result or Wilcoxon Rank-Sum test result for comparison of study characteristics by publication year, as appropriate

^c^Within the subset of trials reporting adjusted analyses, 95% of trials from 2014 vs. 85% of those from 2009 pre-specified adjustments (*p* = 0.0045)

Trials published in the later time period (2014) were less likely to be cluster-randomized (4% vs. 11%; *p* = 0.0297), more likely to report adjusted analyses (whether it be alone or alongside unadjusted analysis result; 87% vs. 79%, *p* = 0.0100), and more likely to have pre-specified adjustment in analyses (95% vs. 85%; *p* = 0.0045).

### Covariate involvement in the design

Table 2 presents trial characteristic summary statistics and individual logistic regression model results grouped by whether covariates were involved in a treatment allocation scheme (outcome #1 from Section 2.2) and further grouped by whether the allocation method was covariate-adaptive (outcome #2 from Section 2.2). Multicenter (82% vs. 63%, *p* = 0.0212), superiority (83% vs. 78% for non-inferiority and 55% for other study types, *p* = 0.0140) studies with fewer arms (*p* = 0.0246) tended to involve baseline variables in the treatment allocation scheme. In addition, longer study length (*p* = 0.0158; OR for one-year increase in length: 1.2 [1.0, 1.4]) and nature of primary outcome (*p* = 0.0054) were associated with covariate involvement in the design phase of these clinical trials. Time-to-event outcomes were most likely to involve covariates in allocation [89%]. Increasing number of covariates involved in the allocation scheme corresponded to an increased likelihood of adaptive-method use (*p* < 0.0001; OR: 4.9 [3.0, 8.1]), and there was a marginal relationship with length of study (*p* = 0.0403; OR for a one-year increase in length: 1.1 [1.0, 1.2]). Interestingly, as study initiation year progressed, fewer trials utilized the covariate-adaptive methods of allocation (*p* = 0.0066), with 31% of trials initiated before 2000 reporting covariate-adaptive method use and just 7% of those initiated in the year 2010 or later reporting adaptive methods.

**Table 2:** Covariate Involvement in the Design of Published RCTs ^a^

	Covariates Involved in Allocation (*N* = 298)					Covariate-Adaptive Method (*N* = 237) ^b^				
	No		Yes		*p*-value	No		Yes		*p*-value
	N	%	N	%		N	%	N	%	
Type of Study					0.0140					0.4694
Superiority	43	17	212	83		182	88	26	13	
Non-Inferiority	5	22	18	78		14	78	4	22	
Other	9	45	11	55		9	82	2	18	
Number of Arms					0.0246					0.7461
2	40	17	196	83		167	87	26	13	
3	5	21	19	79		16	84	3	16	
4	7	27	19	73		15	83	3	17	
5 or more	5	42	7	58		7	100	0	0	
Multicenter Study					0.0212					0.9843
No	9	38	15	63		13	87	2	13	
Yes	48	18	226	82		192	86	30	14	
Number of Centers, median (IQR)	12 (2–51)		24 (7–76)		0.8676	23 (7–76)		26 (6–64)		0.3399
Number of Subjects, median (IQR)	546 (200–1032)		584 (250–1442)		0.3601	580 (255–1369)		613 (188–1389)		0.5002
Length of Study, Years, median (IQR)	3 (2–4)		3 (2–5)		0.0158	3 (2–5)		3 (3–7)		0.0403
Months of Subject Follow-up, median (IQR)	6 (3–18)		12 (6–22)		0.1825	12 (6–19)		12 (6–36)		0.5449
Study Initiation Year					0.0889					0.0066
Unable to Determine	1	20	4	80		3	75	1	25	
Before 2000	1	6	16	94		11	69	5	31	
2000–2004	13	17	62	83		49	82	11	18	
2005–2009	23	19	100	81		87	89	11	11	
2010 and later	19	24	59	76		55	93	4	7	
Primary Outcome					0.0054					0.4861
Continuous	23	26	66	74		55	86	9	14	
Binary	19	17	94	83		82	89	10	11	
Time-to-Event	9	11	75	89		64	85	11	15	
Other	6	50	6	50		4	67	2	33	
Number of Covariates (involved in randomization algorithm)										<.0001
1	Not applicable for this outcome.					93	98	2	2	
2						78	93	6	7	
3						30	77	9	23	
4						4	40	6	60	
5 or more						0	0	9	100	

^a^*P*-values correspond to logistic regression results

^b^This subset of 237 studies includes those studies clearly involving either stratified block method (*N* = 205) or an adaptive method (*N* = 32). Refer to Table 1 (205 + 32 studies using stratified block method or covariate-adaptive method, respectively)

### Adjustment in analyses

Table 3 presents similar information to Tables 1 and 2 for presence of adjusted analyses (outcome #3 in Section 2.2, either adjusted alone or both unadjusted and adjusted) and pre-specification of this adjustment (outcome #4 in Section 2.2). Covariate involvement in treatment allocation (*p* = 0.0100; 86% vs. 72%) and increasing number of covariates (*p* = 0.0312; OR for each additional covariate: 1.6 [1.0,2.6]) were both associated with adjusted analyses. Of note, we cannot assume that the same variables involved in adjustment were the same as those in the design, as data collection did not track the specific variables. Finally, likelihood of pre-specified adjustment increased as time progressed (*p* = 0.0029; OR: 2.1 [1.3, 3.5]), with 97% of trials initiated in 2010 or after pre-specifying adjustment compared with 69% of those initiated before 2000.

**Table 3:** Presence and Pre-specification of Adjusted Analyses in Published RCTs ^a^

	Adjusted Analyses Present (N = 298)					Pre-Specified Adjustment (*N* = 249) ^b^				
	No		Yes		*p*-value	No		Yes		*p*-value
	N	%	N	%		N	%	N	%	
Type of Study					0.6856					0.3791
Superiority	40	16	215	84		19	9	196	91	
Non-Inferiority	5	22	18	78		1	6	17	94	
Other	4	20	16	80		3	19	13	81	
Number of Arms					0.4884					0.3666
2	37	16	199	84		19	10	180	90	
3	5	21	19	79		3	16	16	84	
4	4	15	22	85		1	5	21	95	
5 or more	3	25	9	75		0	0	9	100	
Multicenter Study					0.0865					0.0458
No	7	29	17	71		4	24	13	76	
Yes	42	15	232	85		19	8	213	92	
Number of Centers, median (IQR)	8 (3,32)		25 (7,77)		0.1562	10 (2,63)		28 (8,77)		0.3187
Number of Subjects, median (IQR)	449 (124,1030)		580 (250,1489)		0.6507	700 (179,1408)		568 (267,1502)		0.6488
Length of Study, Years, median (IQR)	3 (2,4)		3 (2,5)		0.0996	3 (2,6)		3 (2,5)		0.6060
Months of Subject Follow-up, median (IQR)	6 (3,14)		12 (5,24)		0.1011	12 (3,24)		12 (6,23)		0.6069
Study Initiation Year					0.7281					0.0029
Unable to Determine	3	60	2	40		0	0	2	100	
Before 2000	1	6	16	94		5	31	11	69	
2000–2004	12	16	63	84		7	11	56	89	
2005–2009	22	18	101	82		9	9	92	91	
2010 and later	11	14	67	86		2	3	65	97	
Primary Outcome					0.0526					0.2586
Continuous	12	13	77	87		6	8	71	92	
Binary	27	24	86	76		11	13	75	87	
Time-to-Event	8	10	76	90		4	5	72	95	
Other	2	17	10	83		2	20	8	80	
Covariates Involved in Allocation					0.0100					0.1980
No	16	28	41	72		6	15	35	85	
Yes	33	14	208	86		17	8	191	92	
Number of Covariates					0.0312					0.2949
N/A	16	28	41	72		6	15	35	85	
1	19	20	76	80		6	8	70	92	
2	8	9	78	91		5	6	73	94	
3	6	15	34	85		2	6	32	94	
4	0	0	11	100		3	27	8	73	
5 or more	0	0	9	100		1	11	8	89	
Baseline Test for Significant Differences					0.3033					0.7216
Unable to Determine	2	40	3	60		0	0	3	100	
No	30	18	137	82		12	9	125	91	
Yes	17	13	109	87		11	10	98	90	

^a^*P*-values correspond to logistic regression results

^b^This subset of 249 studies includes those studies reporting either adjusted analyses only (*N* = 87) or both unadjusted and adjusted (*N* = 162)

**Fig. 2:**
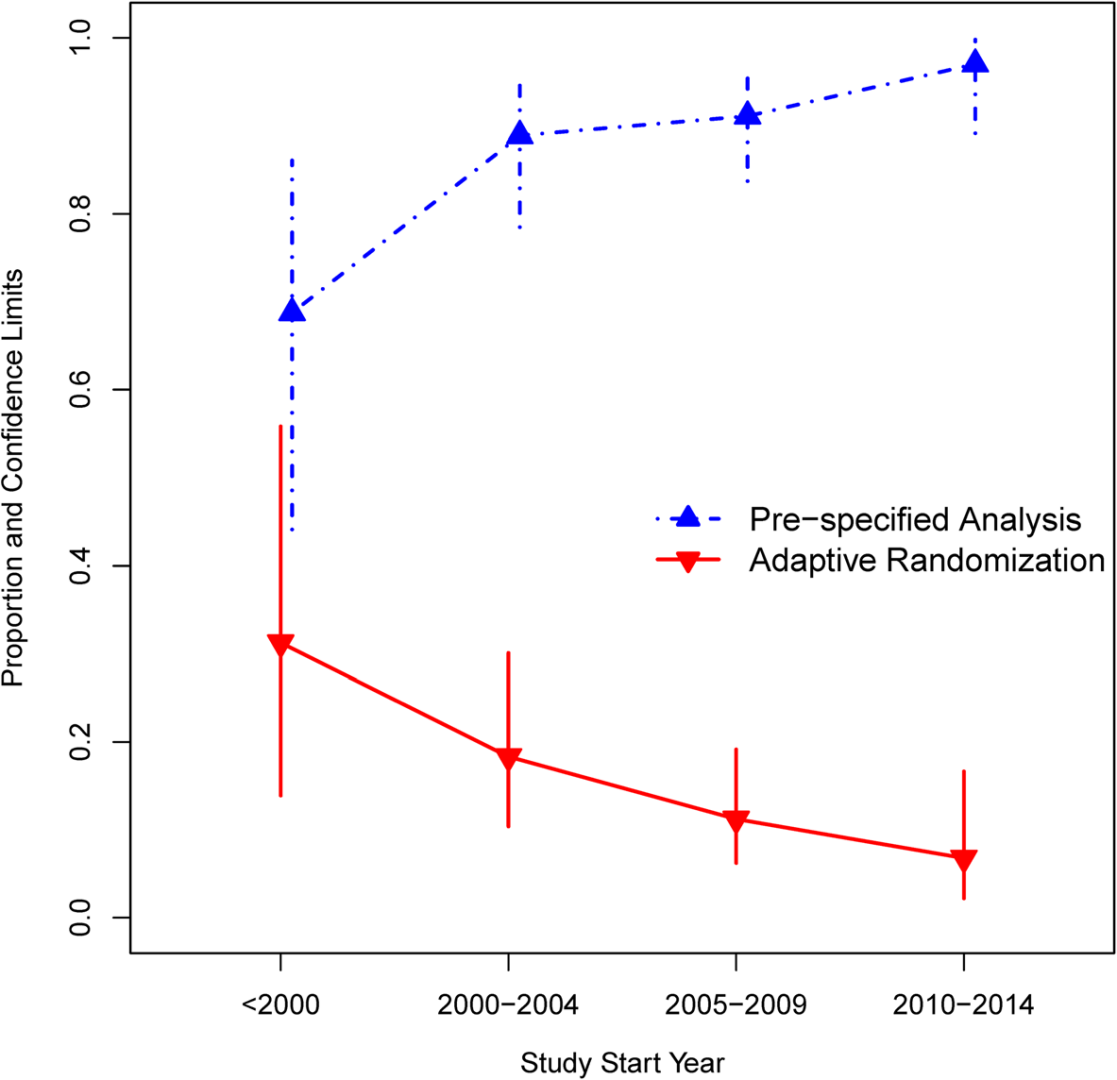
Trials Reporting use of Adaptive Allocation Techniques vs. Pre-specified Analyses over Time. For later study initiation years, trial results were more likely to report pre-specified adjusted analyses. On the contrary, as study start year increased, articles were less likely to report utilization of adaptive allocation techniques over time. We illustrate the proportion along with 95% confidence bands for each grouping of years. Note that the articles reflected here are within the subset of those using covariates in randomization or within the subset of those reporting adjusted analyses

As study initiation year was significantly associated with both pre-specified adjusted analyses and covariate-adaptive allocation in this dataset, we sought to explore this relationship further. Of note, study initiation year was not significantly associated with the other two outcomes of interest (refer to Tables 2 and 3). Figure 2 illustrates sample proportions and 95% confidence limits for pre-specification of adjustment (within the subset of those articles reporting adjustment) and covariate-adaptive method (within the subset of those articles reporting covariate involvement in randomization) reported by study initiation years.

## Discussion

This review of nearly 300 clinical trials in the *NEJM, JAMA, BMJ,* and *Lancet* provides a snapshot of basic trial characteristics and techniques used in handling potentially influential baseline variables in the design and analysis of these studies. During the selected time frames, the typical RCT reported in the four journals explored was: two-armed, multicenter, a superiority trial, lasted for a median of 3 years with median 12 months of follow-up, and employed a stratified block method of treatment allocation with an accompanying analysis that tended to adjust for baseline variables.

As with any study, our review inherently contains several limitations, the major of those being the restricted search to only two six-month time periods from four specific journals. Although we randomly assigned reviewers to each article, we did not stratify on time period nor on journal. The review and data abstraction could have also benefited from a complete independent double-data entry workflow rather than the first / second pass system we chose. Results may also be biased to reflect journal editorial styles, subject matter themes during these time periods, and reviewer preferences. We chose these specific journals to expand upon the previous review by Austin and colleagues [21] and further these journals historically carry high impact factors while reporting results across a diverse group of fields. We chose these specific time points to narrow our search to examine themes before and after updated CONSORT guidelines. Changes incorporated into the 2010 CONSORT revision included improvements in wording and clarity of checklist items, including recommendations [14]. Specifically, edits to Methods checklist items #8b (type of randomization) and 12b (adjusted analyses) would be particularly influential to baseline covariate imbalance. Additional studies expanding the present review into more recent time periods and in topic-specific or a broader range of peer-reviewed journals will provide further insight to the questions we addressed presently, but the present work provides an initial, yet substantial understanding of current practice in trial reporting in light of the recent release of the CONSORT explanation.

In an assessment of the overall progressive nature of clinical trials’ research and practice, positive findings included the dominant use of baseline variables in the design (81%) and analysis (84%) phase and largely pre-specified adjusted analyses (91% among those reporting adjusted analyses), with an increased prevalence of pre-specified analyses over time (Fig. 2, Table 3, *p* = 0.0029). It also would seem logical that covariate involvement in randomization and increasing numbers of covariates would make adjusted analyses more likely. This review did in fact suggest this as 100% of trials with at least four baseline variables involved in allocation presented adjusted analyses. It is comforting and worth noting that as the number of covariates involved in randomization increased, the probability of covariate-adaptive method use also increased (*p* < 0.001; 100% of those with five or more baseline variables involved in randomization employed an adaptive method). We caution the reader and note that these associations may not be inferred as causation as the analyses presented here were exploratory and did not control for potential confounders.

Contrarily, and perhaps disheartening to randomization methodology researchers, despite many shortcomings of the aforementioned stratified blocking scheme, the majority (69%) of trials reported use of the stratified block method. A small proportion (11%) of all reported trials employed a method of covariate-adaptive randomization, illustrating a gap between methodological randomization research and real RCT practice that surprisingly appears to continually widen as time progresses (Fig. 2, *p* = 0.0066). As previously mentioned, adaptive methods with a biasing probability tend to have greater flexibility and better operating characteristics with respect to the number of potential covariates on which one may enforce balance, the prevention of selection bias, and the ability to ensure adequate balance when compared to the simpler stratified block method [11–13, 23–26]. Furthermore, increased complexity in general trial design would inevitably require careful considerations with respect to randomization. Our review suggests an association between increasing number of study arms and decreased probability of covariate involvement in randomization (*p* = 0.0246) with just 58% of trials including five or more arms utilizing baseline variables in allocation in comparison to 83% of trials with two arms. In fact, none of the trials involving five or more arms utilized a covariate-adaptive method. One may argue that with the inherent flexibility of these methods, it would be ideal to employ them more often for complex multi-arm studies. Again, we caution the reader that these findings illustrate associations rather than causation as analyses were exploratory and did not control for potential confounding.

We speculate researchers tend to choose simpler methods of allocation (i.e., simple randomization, stratified blocking schemes) over adaptive methods because they are easier to understand and implement. Investigators often find comfort in the stratified block method as it is familiar and historically the most common method of randomization. Like many methods that are a bit abstract and require more detailed knowledge in theoretical underpinnings, adaptive methods rely on input from a programmer and/or statistician throughout the life of a trial. Principal investigators for RCTs may be reluctant to implement a randomization scheme that is so heavily dependent on statistical personnel, and this is especially true if they lack the understanding regarding its importance in adding efficiency and reducing bias. Furthermore, it was shown in this review that covariate-adaptive method use increased as the number of covariates involved in randomization increased, suggesting that simpler methods of allocation may be preferable because the number of covariates involved in randomization for several studies is limited.

Covariate-adaptive methods oftentimes may not be worth the added benefit in efficiency, but this will depend on each individual study’s objectives, sample size, and logistical/practical constraints; this is especially true for large trials involving diverse populations in which the risk of nontrivial levels of imbalance impacting inference is low. In theory, the randomized nature of RCTs should allow for comparable arms in general. The use of adaptive techniques may be more readily adopted for smaller studies and/or those with large numbers of covariates (as suggested by these data). Further, complex randomization methods will inevitably require more complex analyses, whether it be through adjustment or permutation tests based on randomization methods. This may also serve as a barrier for implementation of these methods since one cannot adjust for all possible covariates and permutation tests, which also add another layer of complexity in interpretation and analyses. However, because this review cannot truly shed light on the reasons for why the gap between randomization methodological research and implementation remains, an area of future research could include a mixed-methods approach, involving focus groups or interviews from trial investigators exploring the motivation for inclusion or exclusion of adaptive randomization methods.

Education and a truly collaborative team science framework in which the study statistician’s role begins with the study’s origin may lessen the gap we illustrate here. Further, with the advent of modern technology and computing power, implementation and programming obstacles should be overcome with minimal effort. As McEntegart pointed out over 10 years ago, “…the pursuit of [baseline covariate] balance could be viewed as a low-cost insurance policy against the likelihood of extreme imbalances, albeit the change of imbalances occurring is low” [24]. Finally, Lin et al. recently independently conducted a very similar review to the one reported herein with similar findings and also provided similar recommendations regarding the use of complex adaptive methods [13].

## Conclusions

Baseline covariate imbalance has potential to affect clinical trial validity and interpretability of results. Ideally, clinical trials would account for baseline variables (1) in the randomization procedure, through covariate adaptive methods that efficiently control imbalance in multiple variables simultaneously, and (2) in analyses via appropriate pre-specified adjustment for these variables. While optimal reporting procedures and pre-specification of adjusted analyses for RCTs tend to be progressively more prevalent over time (97% in trials beginning 2010 or later), we see the opposite effect on reported use of covariate-adaptive randomization methods (31% of trials beginning before 2000 vs. just 7% of those initiated in 2010 or later). All findings together suggest that clinical trial methodology and reporting may be adequate overall, but there still remains a “substantial and confusing variation in handling baseline covariates” in RCTs as previously discussed by Austin et al. [21]. To lessen the confusion, we suggest increased educational efforts regarding the importance of baseline covariates in RCTs, a collaborative team science framework in which the study statistician’s role begins with the study’s origin, and investment in software and personnel time for implementation of these randomization methods.

## Additional file

Additional file 1: Allocation Techniques Review: Data Dictionary Codebook". This file contains a data dictionary (codelists, variable names, field labels, etc.) for our data collection tools used to extract the data from each article our search returned. There were two forms: “Screen” and “Review”. Overall, there were 43 individual data fields. The Screen form contains relevant fields that capture whether each article was retained for further review and data extraction. If not, we captured the reason the article was excluded (field #4, exclude_reason). The Review form contains the data dictionary for each data element extracted from each article reviewed in detail and included in analyses. After review and completion of both forms for all articles, we exported all data in the REDCap database for summarization and analyses reported in this manuscript. (PDF 126 kb)

## Data Availability

The datasets used and/or analyzed during the current study are available from the corresponding author on reasonable request.

## Abbreviations

AY: Amy Yang
BMJ: British Medical Journal
CONSORT: Consolidated Standards of Reporting Trials
HLP: Hannah L. Palac
ICH: International Conference on Harmonization
JAMA: Journal of the American Medical Association
JDC: Jody D. Ciolino
MV: Mireya Vaca
NEJM: New England Journal of Medicine
RCT: Randomized controlled trial
REDCap: Research electronic data capture
