# Supplementary material for: Ideal vs. real: a systematic review on handling covariates in randomized controlled trials
Source: BMC Med Res Methodol. 2019 Jul 3;19:136. doi: 10.1186/s12874-019-0787-8 (PMC6610785; doi:10.1186/s12874-019-0787-8)
Supplement: Supplementary file 1 — Allocation Techniques Review: Data Dictionary Codebook". This file contains a data dictionary (codelists, variable names, field labels, etc.) for our data collection tools used to extract the data from each article our search returned. There were two forms: “Screen” and “Review”. Overall, there were 43 individual data fields. The Screen form contains relevant fields that capture whether each article was retained for further review and data extraction. If not, we captured the reason the article was excluded (field #4, exclude_reason). The Review form contains the data dictionary for each data element extracted from each article reviewed in detail and included in analyses. After review and completion of both forms for all articles, we exported all data in the REDCap database for summarization and analyses reported in this manuscript. (PDF 126 kb) [file 12874_2019_787_MOESM1_ESM.pdf]

Allocation Techniques Review

Data Dictionary Codebook

10/04/2016 2:05pm

	#	Variable / Field Name	Field Label<br>*Field Note*	Field Attributes (Field Type, Validation, Choices, Calculations, etc.)
Instrument: **Screen**				
	1	pmid	PubMed ID:	text
	2	author	First Author:	text
	3	review_yes	Include article in review?	yesno<br>1 Yes<br>0 No
	4	exclude_reason<br>Show the field ONLY if:<br>[review_yes] = '0'	Why was the article excluded?	radio<br>1 Not an RCT<br>2 Review paper<br>3 Editorial<br>4 Commentary<br>88 Other (specify)
	5	exclude_reason_other<br>Show the field ONLY if:<br>[exclude_reason] = '88'	Other (specify)	text
	6	printed	Printed outside of inclusion timeframe?	yesno<br>1 Yes<br>0 No
	7	printed_year<br>Show the field ONLY if:<br>[printed] = '1'	What year was the article originally printed?	text (number)
	8	screen_complete	Complete?	dropdown<br>0 Incomplete<br>1 Unverified<br>2 Complete
Instrument: **Review**				
	9	title	Title:	text
	10	journal	Journal:	radio<br>0 NEJM<br>1 JAMA<br>2 BMJ<br>3 Lancet
	11	year_pub	Publication year:	text (number, Min: 2000, Max: 2015)
	12	size	Number of participants randomized	text (number)

	13	size_analytic	Analytic sample size:	text
	14	arms	Number of arms:	text (number, Min: 2, Max: 5)
	15	studytype	Study type:	radio<br>1 Superiority ("Regular" RCT)<br>2 Non-inferiority (NI)<br>3 Equivalence<br>4 Crossover<br>88 Other (specify)
	16	studytype_other<br>Show the field ONLY if:<br>[studytype] = '88'	Other (specify):	text
	17	cluster	Cluster-randomized?	yesno<br>1 Yes<br>0 No
	18	multicenter	Multicenter study?	yesno<br>1 Yes<br>0 No
	19	multicenter_centers<br>Show the field ONLY if:<br>[multicenter] = '1'	How many centers?	text (number, Min: 2)
	20	year_study	Study start year:	text (number)
	21	length_reported	Was study length reported?	yesno<br>1 Yes<br>0 No
	22	length<br>Show the field ONLY if:<br>[length_reported] = '1'	Study length:<br>*Please indicate units of time (e.g. days, weeks, years).*	text
	23	length_units<br>Show the field ONLY if:<br>[length_reported] = '1'	Study length units:	radio<br>1 Days<br>2 Weeks<br>3 Months<br>4 Years<br>88 Other (specify)
	24	length_units_other<br>Show the field ONLY if:<br>[length_units] = '88'	Other (specify):	text
	25	followup_reported	Was participant follow-up time reported?	yesno<br>1 Yes<br>0 No
	26	followup<br>Show the field ONLY if:<br>[followup_reported] = '1'	Participant follow-up time:<br>*If survival analysis, use the median follow-up time. Round to the nearest whole number. Please*	text (number, Min: 0)

			indicate units of time (e.g. days, weeks, years) below.	
	27	followup_units<br><br>Show the field ONLY if:<br>[followup_reported] = '1'	Participant follow-up units:	radio<br>1 Days<br>2 Weeks<br>3 Months<br>4 Years<br>88 Other (specify)
	28	units_other<br><br>Show the field ONLY if:<br>[followup_units] = '88'	Other (specify):	text
	29	random_title	Randomized in title:	yesno, Required<br>1 Yes<br>0 No
	30	random_scheme	Randomization scheme used:	radio, Required<br>1 Purely random allocation<br>2 Blocked (Permuted or Random block)<br>3 Stratified or Stratified block<br>4 Urn design<br>5 Biased coin design<br>6 Minimization/Covariate adaptive method<br>7 Response adaptive method<br>9 Unable to determine<br>88 Other (specify)
	31	random_scheme_other<br><br>Show the field ONLY if:<br>[random_scheme] = '88'	Other (specify):	text
	32	random_covariates	Were covariates involved in randomization?	radio, Required<br>1 Yes<br>0 No<br>9 Unable to determine
	33	covariate_number<br><br>Show the field ONLY if:<br>[random_covariates] = '1'	How many covariates were involved in randomization?<br>*Include "site" or "center" if listed.*	text (integer, Min: 1)
	34	random_clear	In your opinion, was the reporting on randomization CLEAR?	yesno<br>1 Yes<br>0 No
	35	table1	Did the article include a "Table 1"?	yesno, Required<br>1 Yes<br>0 No

	36	imbalance_test<br>Show the field ONLY if:<br>[table1] = '1'	Baseline test used (see Table 1):	yesno, Required<br>1 Yes<br>0 No
	37	outcome_type	What is the nature of the primary outcome variable?	radio, Required<br>1 Continuous<br>2 Binary<br>3 Time-to-event<br>4 Ordinal/Categorical<br>5 Unable to determine<br>88 Other (specify)
	38	outcome_type_other<br>Show the field ONLY if:<br>[outcome_type] = '88'	Other (specify):	text
	39	imbalance_adj	Type of analysis:	radio, Required<br>0 Unadjusted<br>1 Adjusted<br>2 Both<br>9 Unable to determine
	40	adj_reason<br>Show the field ONLY if:<br>[imbalance_adj] = '1' or [imbalance_adj] = '2'	Reason for adjustment:	radio<br>1 Pre-specified<br>2 Data driven - Lack of balance<br>3 Data driven - Potential confounding<br>9 Unable to determine<br>88 Other (specify)
	41	adj_reason_other<br>Show the field ONLY if:<br>[adj_reason] = '88'	Other (specify):	text
	42	comments	Comments:	notes
	43	review_complete	Complete?	dropdown<br>0 Incomplete<br>1 Unverified<br>2 Complete
